# Fabrication of Mechanically Alloyed Super Duplex Stainless Steel Powder-Modified Carbon Paste Electrode for the Determination of Methylene Blue by the Cyclic Voltammetry Technique

**DOI:** 10.1021/acsomega.3c09163

**Published:** 2024-02-21

**Authors:** Rayappa Shrinivas Mahale, Vinaykumar Rajashekar, Shamanth Vasanth, Sharath Peramenahalli Chikkegowda, Shashanka Rajendrachari, Vutukuru Mahesh

**Affiliations:** † School of Mechanical Engineering, REVA University, Bengaluru 560064, Karnataka, India; ‡ Department of Mechanical Engineering, Jain College of Engineering and Research, Udyambag, Belagavi 590008, Karnataka, India; § Department of Electronics and Communication Engineering, Nitte Meenakshi Institute of Technology, Bengaluru 560064, Karnataka, India; ∥ Department of Electronics and Communication Engineering, Faculty of Engineering and Technology, JAIN Deemed to be University, Bengaluru 562112, Karnataka, India; ⊥ Department of Metallurgical and Materials Engineering, BARTIN University, 74100 Bartin, Turkey; # Mechanical Engineering, SR University, Warangal 506371, Telangana, India

## Abstract

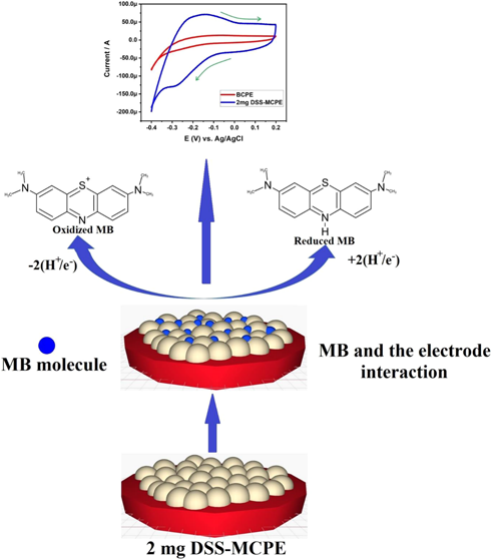

Alloys with an equal balance of ferrite and austenite provide super duplex stainless steel (DSS) with enhanced strength and corrosion resistance. This study utilized mechanical alloying to produce nanostructured super duplex stainless steel powders for the identification of methylene blue dye in wastewater. High-energy particle grinding was employed to create the SAF-2507 DSS powders. To electrochemically oxidize methylene blue dye in wastewater, a modified carbon paste electrode (DSS-MCPE) was developed. Methylene blue, a water-soluble cationic colorant extensively used in the paper, pulp, and textile industries, poses a threat to human health and water supplies when improperly disposed of. DSS-MCPE demonstrated a significant current response, indicating its capability to detect methylene blue dye in a pH range of 6–8. The experiment revealed that 2 mg of DSS-MCPE produced a maximum current response of 72.22 μA, facilitating the effective electrooxidation of methylene blue dye in wastewater. Furthermore, the investigation demonstrated that the active surface area of the 2 mg of DSS-MCPE (0.478 cm^2^) was greater than that of the bare carbon paste electrode (BCPE) (0.054 cm^2^). The increased active surface area was correlated with an enhanced current response. The strong interaction between methylene blue molecules at the interface of the produced 2 mg of DSS-MCPE contributed to the observed increase in anodic current across methylene blue concentrations ranging from 0.1 to 0.6 mM.

## 1 Introduction

Super duplex stainless steels are renowned for their exceptional performance in highly corrosive chloride conditions, exhibiting excellence in stress corrosion cracking resistance, resistance to pitting, and corrosion fatigue. This positions them as standout materials among other advanced stainless steel alloys. A key characteristic that distinguishes super duplex stainless steel is its nearly equal composition of ferrite (α-BCC) and austenite (γ-FCC). The phase balance in chemical composition and thermomechanical behaviors is intimately related to this equilibrium. Notably, super duplex stainless steel contains less nickel (Ni) than standard austenitic grades while maintaining excellent strength and remarkable malleability. These steels are the material of choice for applications demanding outstanding performance in harsh and corrosive conditions due to their unique combination of characteristics.^1,2^ An additional advantage of super duplex stainless steel is its lower Ni concentration, making it suitable for use as a biomaterial in situations where patients might experience hypersensitivity due to higher Ni content. Super duplex stainless steels find application in orthopedic devices such as Harrington rods for scoliosis treatment, addressing abnormal lateral curvature of the spine thanks to their increased biocompatibility. Industries such as pulp and paper, mineral processing, marine, and structural engineering greatly benefit from the application of super duplex stainless steels.^3^

Various processes, including hot forming, solution annealing, powder forging, equal channel angular pressing, and mechanical alloying, can be employed to produce super duplex stainless steels. Among these methods, mechanical alloying stands out as the most practical and economical means of creating materials on the nanoscale. Mechanical alloying involves the use of high impact force, leading to phase transition, microstructure refinement, repetitive cold welding, severe plastic deformation, and powder particle fracture. Finer microstructures achieved through mechanical alloying offer unique qualities such as increased surface area, oxidation resistance, and strength.^4−6^ Mechanically alloyed super duplex stainless steels find applications in various electrochemical sensing applications, including the detection of heavy metals in environmental pollutants, monitoring of chemical and biological parameters, identification of hazardous substances in food, and the diagnosis of bacteria and viruses, among others.^7−13^ In this study, SAF-2507 super duplex stainless steel nanoparticles were ball-milled for 20 h to produce a modified carbon paste electrode (DSS-MCPE) for the detection of methylene blue in wastewater. The research conducted on creating super duplex stainless steels by mechanical alloying is outlined in Table 1.

**Table 1:** Studies Related to the Fabrication of DSS by Mechanical Alloying

material/composition	milling process parameters	crystallite/grain morphology and particle size	references
UNS S32520 DSS	attritor, 500 rpm mill speed, 50:1 ball-to-powder ratio (BPR), and 15 h of milling	uneven grain structure with an average particle size of around 20 μm	(14)
duplex and ferritic stainless steel	Fritsch Pulverisette Planetary Grinder, BPR 6:1, 300 rpm, 40 h grinding period	round particles having an austenitic stainless steel crystallite size of 9 nm and a ferritic stainless steel crystallite size of 11 nm.	(15)
duplex and ferritic stainless steel	planetary mill with BPR ratios of 6:1 and 12:1, main shaft speed of 275 rpm, jar speed variation of 620–726 rpm, and up to 10 h of ball milling	amorphous particles having a crystallite size of 10 nm for ferritic stainless steel and 9 nm for duplex stainless steel.	(16)
duplex and ferritic stainless steel	Fritsch Pulverisette Planetary Grinder, BPR 6:1, 300 rpm, 40 h grinding period	spheres with crystallite sizes of 7 nm for ferritic stainless steel and 8 nm for duplex stainless steel	(17)
duplex stainless steel	275 rpm, dual drive planetary mill, ball-to-powder ratio of 6:1, and 10 h ball milling	spherical particles, typically measuring between 5 and 10 μm in size	(18)
UNS S31803 duplex stainless steel	planetary ball mill with a 15:1 ball-to-powder ratio, 350 rpm, and a 20 h ball milling cycle	particles that are irregular, having an average size of around 175.6 μm	(19)
duplex and ferritic stainless steel	high-energy planetary ball mill with a 6:1 ball-to-powder ratio, 300 rpm, and a 10 h ball milling cycle	particles that are spherical and have a crystallite size of 6 nm and a particle size of around 3 μm	(20)
SAF-2507 DSS	Retsch PM-100 planetary ball mill with a 5:1 ball-to-powder ratio, 250 rpm, and a 20 h ball milling cycle	spherical particles having an 88 nm crystallite size	current paper

Methylene blue (MB), a water-soluble cationic colorant, is utilized in the treatment of conditions such as methemoglobinemia and malaria. In hematology, MB is employed in staining techniques like Wright’s and Jenner’s stains to distinguish between different blood cell types. The stains aid in the differentiation of various white blood cell types based on their shape and color. In microbiology, MB is used to visualize pathogen genetic material under a microscope. The acidic nucleic acid of a pathogen reacts with MB, producing a redox reaction that transforms RNA or DNA into a recognizable blue hue and facilitates easier observation. Various industries, including paper, leather, and textiles, use methylene blue as a coloring agent. However, the substantial discharge of MB dye into surface and groundwater by these industries poses environmental challenges, leading to health problems and detrimental impacts on ecosystems. Popular techniques for detecting methylene dye in wastewater include surface adsorption, membrane filtration, coagulation, and flocculation. Activated carbon is often employed as a sorbent in these methods. Cyclic voltammetry, a technique widely used to investigate the electrochemical behavior of substances, is used to ascertain the electrochemical oxidation of methylene blue in wastewater. This technique offers details about the chemical’s potential, desired features, and structure, known for its affordability, simplicity, sensitivity, and speed, making it particularly useful in the biomedical industry.^21−22,23,24,25,26,27,28,29,30,31^

In this context, a carbon paste electrode modified with SAF-2507 super duplex stainless steel (DSS) nanopowder was developed to investigate the electrochemical oxidation of methylene blue in wastewater using cyclic voltammetry. Alloys, such as super duplex stainless steel, are favored in such studies due to their superior stability, resistance to corrosion, increased conductivity, and favorable electrochemical properties. Prior research employing cyclic voltammetry to explore the electrochemical behavior of methylene blue in wastewater is summarized in Table 2.

**Table 2:** Studies Reported by Various Researchers to Determine the Presence of MB by Cyclic Voltammetry Approach

type of the electrode	experimental specifications	observations	references
platinum screen-printed electrode	at a Pt SPE with a pH of 7, and in the context of a reversible reaction, the observed potential difference between the oxidation and reduction peaks is recorded at 59 mV.	cyclic voltammetry (CV) is a useful tool for conducting electrochemical investigations of methylene blue redox reactions on screen-printed electrodes (SPEs). Moreover, optical and Raman spectroscopy can be used in conjunction with the investigation of these processes to provide a thorough spectroscopic examination.	(32)
HEA-MCPE	pH = 6–7.6 and 1 mM MB concentration	the behavior of MB electrochemically at different scan speeds, pH values, and concentrations of MB.	(33)
NH_2_-*f*MWCNTs-GCE	pH = 3–9 and 0.1 M concentration	in order to identify methylene blue in aqueous solutions, the NH2-fMWCNTs-GCE was developed. The created sensor’s performance was assessed using square-wave voltammetry, cyclic voltammetry, and electrochemical impedance spectroscopy.	(34)
mercaptoacetic acid self-assembled monolayer DNA-modified gold electrode	pH = 6 and 1.5 × 10^–4^ mol/L MB concentration	using methylene blue as an electrochemical indicator, an electrochemical DNA biosensor derived from a genetically engineered organism was developed and employed to directly detect PCR products derived from the NOS gene.	(35)
gold electrode	pH = 7.4 and concentration from 100 nM to 1 μM	the model shows a substantial correlation between the chemical makeup of blocking self-built monolayers and the proton supplied during the first reduction step of methylene blue.	(36)
glassy carbon electrode	pH = 7 and concentration range of 0.000333–2.28 mM.	using GO-NMB nanocomposite, an electrochemical sensor was suggested for use in H_2_O_2_ measurement.	(37)
glassy carbon electrode	pH = 8.2 and concentration range from 1 × 10^–7^ to 5 × 10^–5^ M	because of the dye’s competitive displacement from DNA molecules and enhanced reactivity in the redox process at the electrode, the methylene blue peak currents rose as the doxorubicin concentration increased.	(38)
glassy carbon electrode	pH = 7 and 2.5 × 10^–6^ mol L^–1^ concentration	using methylene blue (MB) as the electroactive probe, this modified electrode was successfully used to identify DNA damage that occurred indirectly.	(39)
MB-doped polyimide-modified glassy carbon electrode	pH = 7 and concentrations from 0.1 to 3 mM.	for the measurement of ascorbic acid, the MB-doped polyimide-modified glassy carbon electrode sensor exhibits high selectivity, quick response, and extended life span.	(40)
glassy carbon electrode	pH = 7 and concentrations from 0.099 to 69.51 μM	the developed PMb/ZnO NPs/GCE electrode worked well for measuring the amount of vitamin B12 in supplements that were sold commercially.	(41)
gold–carbon electrode	pH = 4.3 and 1.0 mM concentration	the constructed sensor implies that it may be used in textile wastewater treatment to remove synthetic dyes.	(42)
			current paper

## 2 Materials and Methods

### 2.1 Preparation of SAF-2507 DSS Nanopowders Using a High-Energy Planetary Ball Mill

The SAF-2507 super duplex stainless steel (DSS) powder, with an average particle size ranging from 45 to 60 μm, is acquired from Sandvik Osprey Ltd. in the U.K. The powder undergoes a 20 h ball milling process conducted in a Retsch PM-100 high-energy planetary ball mill. In this milling operation, the ball-to-powder ratio is set at 5:1, and the mill operated at a speed of 250 rpm. The milling jar, with a capacity of 500 mL, contained stainless steel balls measuring 10 mm in diameter, forming the milling medium. To prevent overheating of the milling container, the ball mill is halted for 30 min every 2 h.

### 2.2 Fabrication of BCPE and DSS-MCPE

A bare carbon paste electrode (BCPE) was formulated through manual blending of graphite powder and silicone oil in a 70:30 ratio using a pestle and mortar for a duration of 30 min.^44,45^ Employing a similar methodology, DSS powders were introduced into the aforementioned composition through hand grinding, leading to the creation of an electrode termed DSS-MCPE. Different concentrations of DSS-MCPE were achieved by adding 2, 4, 6, 8, and 10 mg of DSS to the BCPE composition. The assembly details of the prepared carbon paste electrodes and the electrode system used have been comprehensively elucidated by the authors in their previous publications.^46−48^

## 3 Results and Discussion

### 3.1 XRD Phase Analysis

**Figure 1:**
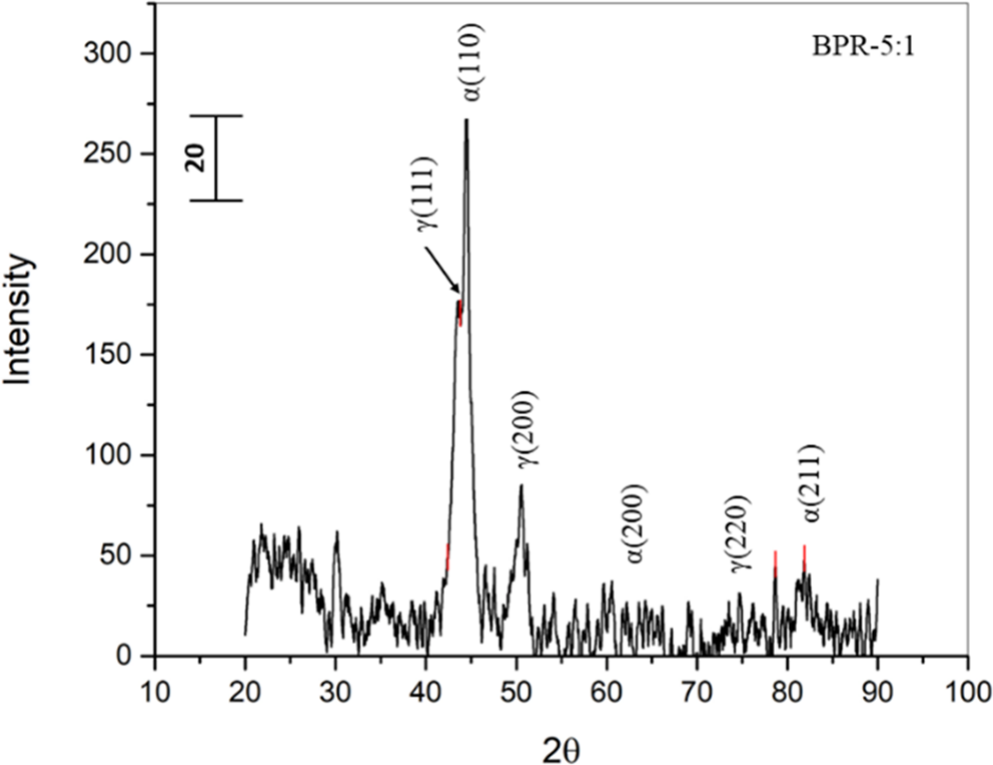
XRD spectra of 20 h ball-milled SAF-2507 DSS powder with 5:1 BPR.

The X-ray diffraction (XRD) pattern presented in Figure 1 illustrates the impact of a 20 h ball milling process on SAF-2507 super duplex stainless steel (DSS) powder. In its initial state, the powder particles exhibited a spherical morphology with an average size ranging from 35 to 60 μm. Postmilling, significant alterations are evident. Specifically, the (110) peak experiences a shift to a lower angle, indicating the emergence of the austenitic phase (111). The initially sharp XRD peaks, indicative of crystallinity, undergo widening after 20 h of ball milling. This broadening is attributed to the continuous impact of milling media on the powder particles, resulting in their fracturing. The widened peaks signify the induction of amorphous behavior in the powder, leading to the formation of a solid solution of metals within the alloy.

The observed peak broadening is linked to various milling-induced processes encompassing structural flaw development, amorphization, and a reduction in particle size. Together, these processes contribute to the broadening of the diffraction peaks, signifying crucial transformations such as the diffusion of Cr and Ni atoms into the Fe lattice. This diffusion initiates a phase transition from ferrite to austenite, highlighting the dynamic structural changes occurring during the ball milling process.

### 3.2 Powder Morphology Studies

**Figure 2:**
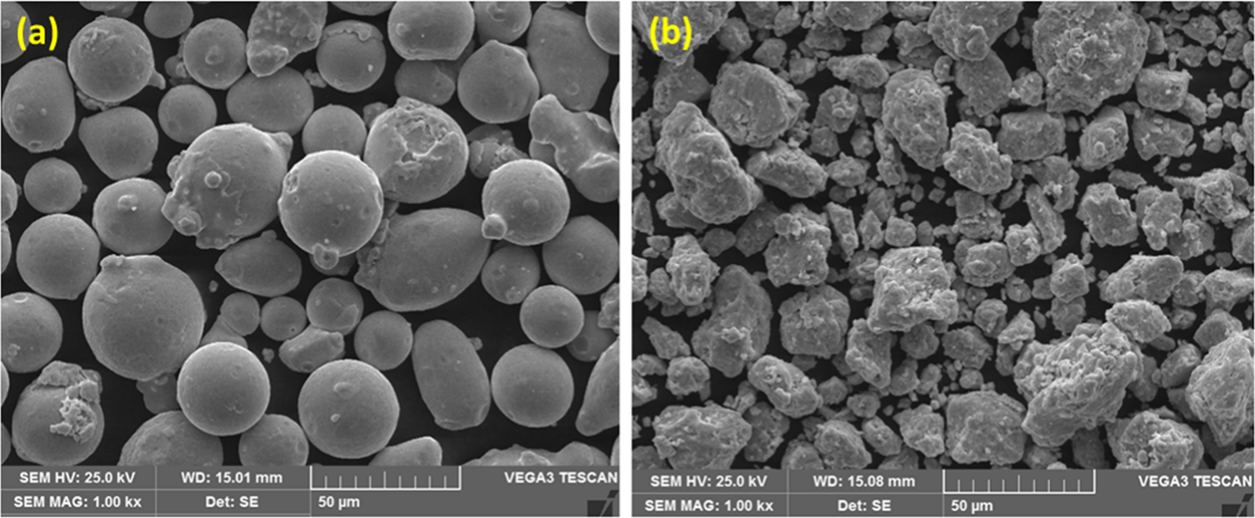
(a) SEM micrograph of as received and (b) 20 h ball-milled SAF-2507 DSS powder.

In Figure 2a, a scanning electron microscopy (SEM) micrograph illustrates the as-received powder featuring large, spherical particles. Following 5 h of ball milling, the ductile nature of iron (Fe) contributes to the flattening of particles. After 20 h of ball milling, as depicted in Figure 2b, the powder particles undergo refinement, with nickel (Ni) and chromium (Cr) atoms assimilating into the Fe lattice, forming an alloy. The particles also exhibit a work-hardened structure. The optimization of milling time is crucial, and in this study, 20 h is identified as the optimal duration. Prolonged milling could lead to elevated contamination levels, resulting in the formation of undesired phases.^43^ The resulting powder particles in Figure 2b display an uneven shape, underscoring the impact of the milling time on powder morphology. Other milling process parameters, such as ball-to-powder weight ratio, milling environment, and milling media type, can also influence powder morphology. The study emphasizes the significance of a 20 h ball milling time as the optimal parameter for achieving refined particles and enhanced surface area.

**Figure 3:**
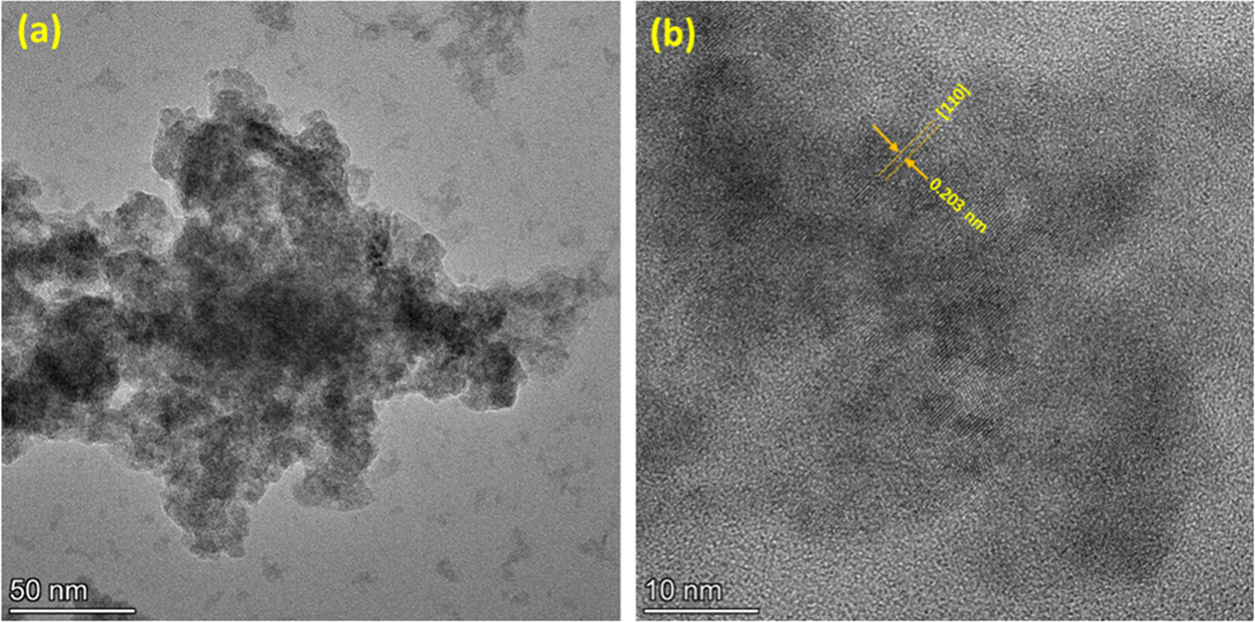
(a) TEM and (b) HR-TEM micrographs of 20 h ball-milled SAF-2507 DSS powder.

For high-resolution transmission electron microscopy (HRTEM) analysis, the size and lattice characteristics of the 20 h ball-milled SAF-2507 DSS powder are precisely determined. In Figure 3a, the transmission electron microscopy (TEM) micrograph reveals the presence of amorphous clusters comprising nanocrystalline particles of approximately 50 nm in size. Figure 3b presents an HRTEM micrograph, where the distance between successive parallel planes of atoms (*d*-spacing) measures 0.203 nm. The micrograph in Figure 3a distinguishes between microsized particles in black and nanocrystals in gray areas.

### 3.3 Electrochemical Determination of MB

#### 3.3.1 Determining the Optimum Modifier Concentration for the Carbon Paste Electrode

**Figure 4:**
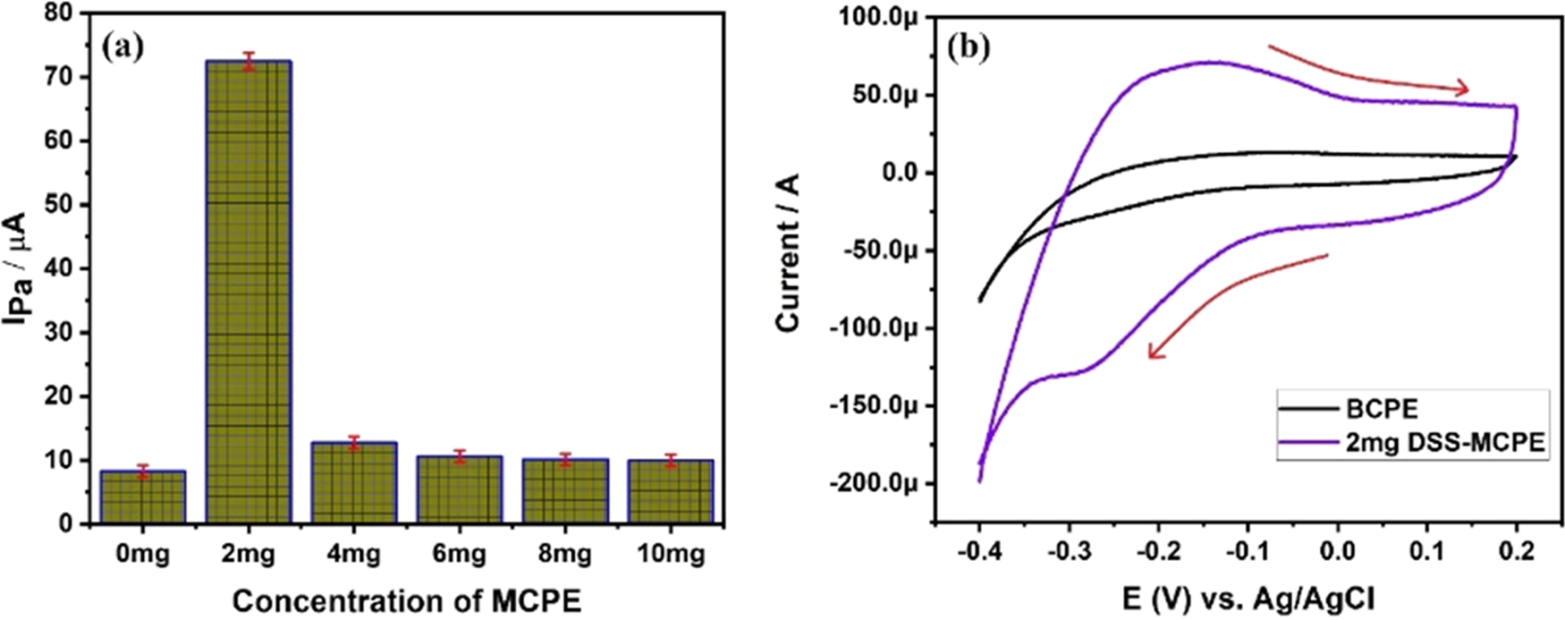
(a) Graph of oxidation peak current recorded at different concentrations of DSS-MCPE. (b) Cyclic voltammogram (CV) of *I*_pa_ of BCPE and 2 mg of DSS-MCPE at 0.1 mM MB solution at pH 8 and a scan rate of 0.1 V/s respectively.

The determination of the maximum current response is significantly dependent on the concentration of the modifier used. Therefore, it is essential to explore the electrochemical oxidation of MB at different concentrations of DSS-MCPEs and select the electrode with the highest current response for further analysis. Various concentrations (0, 2, 4, 6, 8, and 10 mg) of DSS-modified MCPEs were prepared, and cyclic voltammograms were recorded for each concentration. The corresponding oxidation peak current plot is presented in Figure 4a. The plot indicates that the 2 mg of DSS-MCPE exhibited the maximum anodic peak current (*I*_pa_) of 72.22 μA during the electrochemical oxidation of 0.1 mM MB at pH 8. In contrast, BCPE showed an Ipa value of only 8.26 μA. Figure 4a suggests that 2 mg of DSS-MCPE demonstrated higher current sensitivity compared to 4, 6, 8, and 10 mg DSS-MCPEs. Consequently, 2 mg of DSS-MCPE was selected for further electrochemical studies, including the effect of pH variation, scan rate, and analyte concentrations.

**Figure 5:**
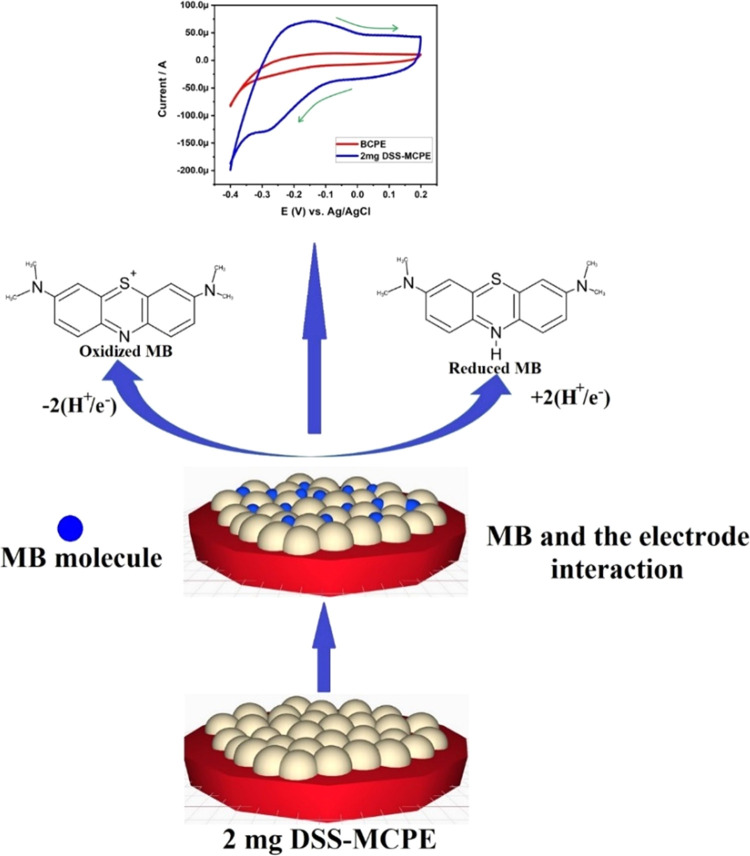
Possible mechanism of electrochemical redox of MB at the fabricated 2 mg of DSS-MCPE.

Figure 4b illustrates the cyclic voltammetric graph of BCPE and 2 mg of DSS-MCPE, highlighting substantial differences in their Ipa. Modification of the carbon paste electrode is crucial for obtaining maximum current response, and the anodic peak current of 2 mg DSS-MCPE is at least 9 times that of BCPE. This significant increase in the current response of the 2 mg DSS-MCPE is attributed to the enhanced surface area, which, in turn, increases the number of active sites. This augmentation promotes the mobility of electrons between the electrode and the electrolyte containing the analyte. The possible mechanism of the electrochemical redox of MB at the fabricated 2 mg of DSS-MCPE is shown in Figure 5. To delve deeper into this, we calculated the electrode active surface area of both BCPE and 2 mg of DSS-MCPE using the Randles–Sevcik equation,^49,50^ as outlined below:
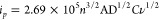
1The electrode active surface areas for both BCPE and 2 mg of DSS-MCPE were successfully calculated, resulting in values of 0.054 and 0.478 cm^2^, respectively. This indicates a significant increase in the surface area of the electrode, correlated with the enhanced current response. Another contributing factor to the increased current response is the composition of the duplex stainless steel powders utilized. The primary components of these powders are Fe, Cr, and Ni, all of which belong to d-block elements. Numerous researchers have reported that d-block elements exhibit excellent electrocatalytic activity, particularly in the determination of various bioactive compounds and organic dyes.^51,52^

#### 3.3.2 Investigating the Effect of pH on the Electrochemical Oxidation of MB

**Figure 6:**
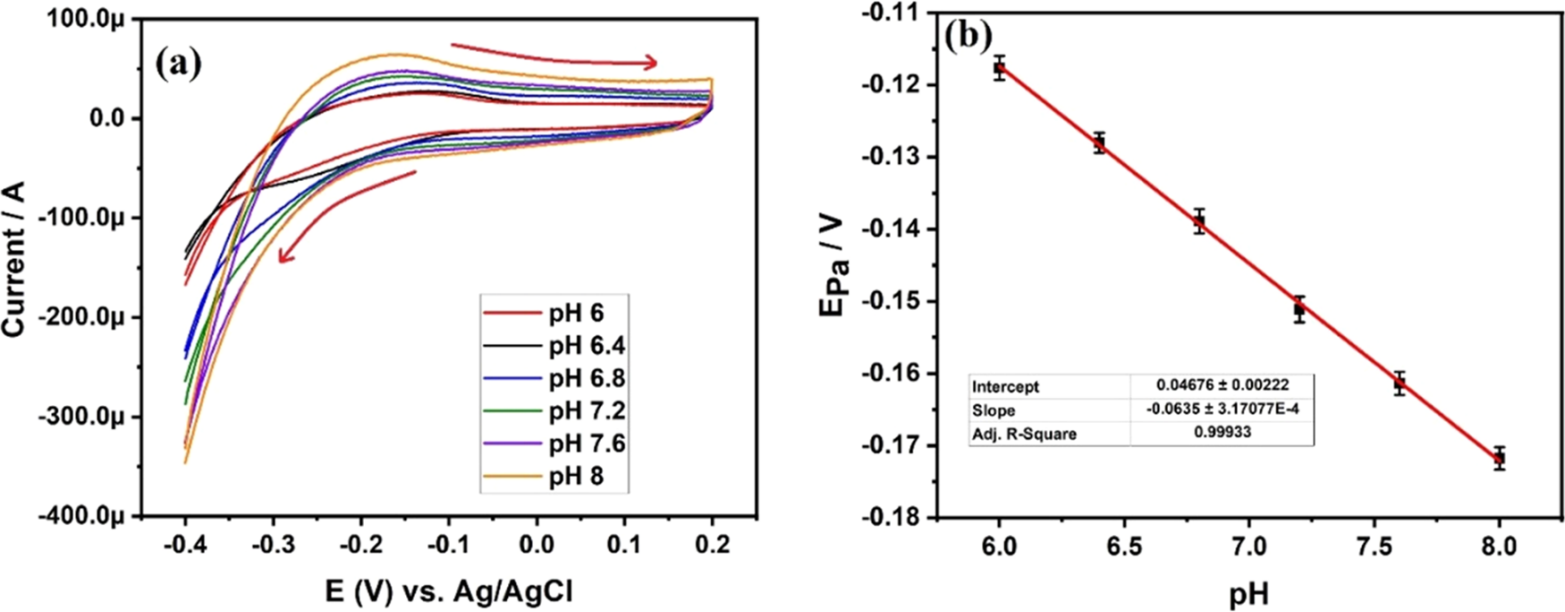
(a) CV curves of 2 mg of DSS-MCPE in 0.1 mM MB at pH range between 6 and 8. (b) Plot of pH vs Epa at 0.1 mM MB.

Achieving optimal pH is a critical factor that influences the electrode’s sensitivity, stability, and selectivity, thereby enhancing the electron interaction between the electrode and the analyte. In Figure 6a, the cyclic voltammogram curve of 2 mg of DSS-MCPE in 0.1 mM MB is depicted at various pH solutions (ranging from 6 to 8) in a phosphate buffer solution (PBS). The voltammogram exhibits a proportional increase in the oxidation peak current with rising pH from 6 to 8, suggesting enhanced stability of the analyte MB in a more alkaline medium.

Figure 6b portrays the plot of *E*_pa_ at different pH values, revealing a linear shift of *E*_pa_ toward the negative potential, indicating the participation of protons in the electrochemical oxidation of MB. The plot follows a linear trend, described by the equation (*V*) = 0.4676 – (0.0635) pH (*V*/pH) (*R*^2^ = 0.9993), attesting to an outstanding linear regression coefficient (*R*^2^). This observation supports the notion that the electron transfer during the redox reaction of MB predominantly relies on protonation. While it confirms the involvement of protons, the precise quantity remains uncertain. To address this, we calculated the number of electrons and protons engaged in the electrochemical reaction of MB using the following equation
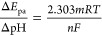
2The calculated number of protons (*m*) involved for the electrochemical reaction is 2.1475, and the value is almost equal to 2. Hence, this confirms the participation of 2 protons and 2 electrons for the electrochemical redox reaction of MB.

Nonetheless, certain researchers, exemplified by Ju et al., have asserted the involvement of only one H+ in the electrode reaction.^53^ In contrast, our investigation suggests that the synergistic action of 2 protons and 2 electrons notably amplifies the current response. Bauldreay and Archer have advocated a similar perspective, positing an augmented number of electrons and protons.^54^ They propose that oxidizing MB at a suitable voltage leads to a cationic reactive radical, serving as a potent electron–proton acceptor on the electrode surface.

Moreover, another study has emphasized the participation of 2 protons in the redox reaction of MB, particularly within the pH range of 5.5–7.4 and exclusively at higher scan rates.^55^ Hence, the selection of an optimal pH level can augment the quantity of protons, thereby boosting electron mobility and current response and fostering a robust interaction between the electrode surface and the analyte.

#### 3.3.3 Investigating the Scan Rate Effect

**Figure 7:**
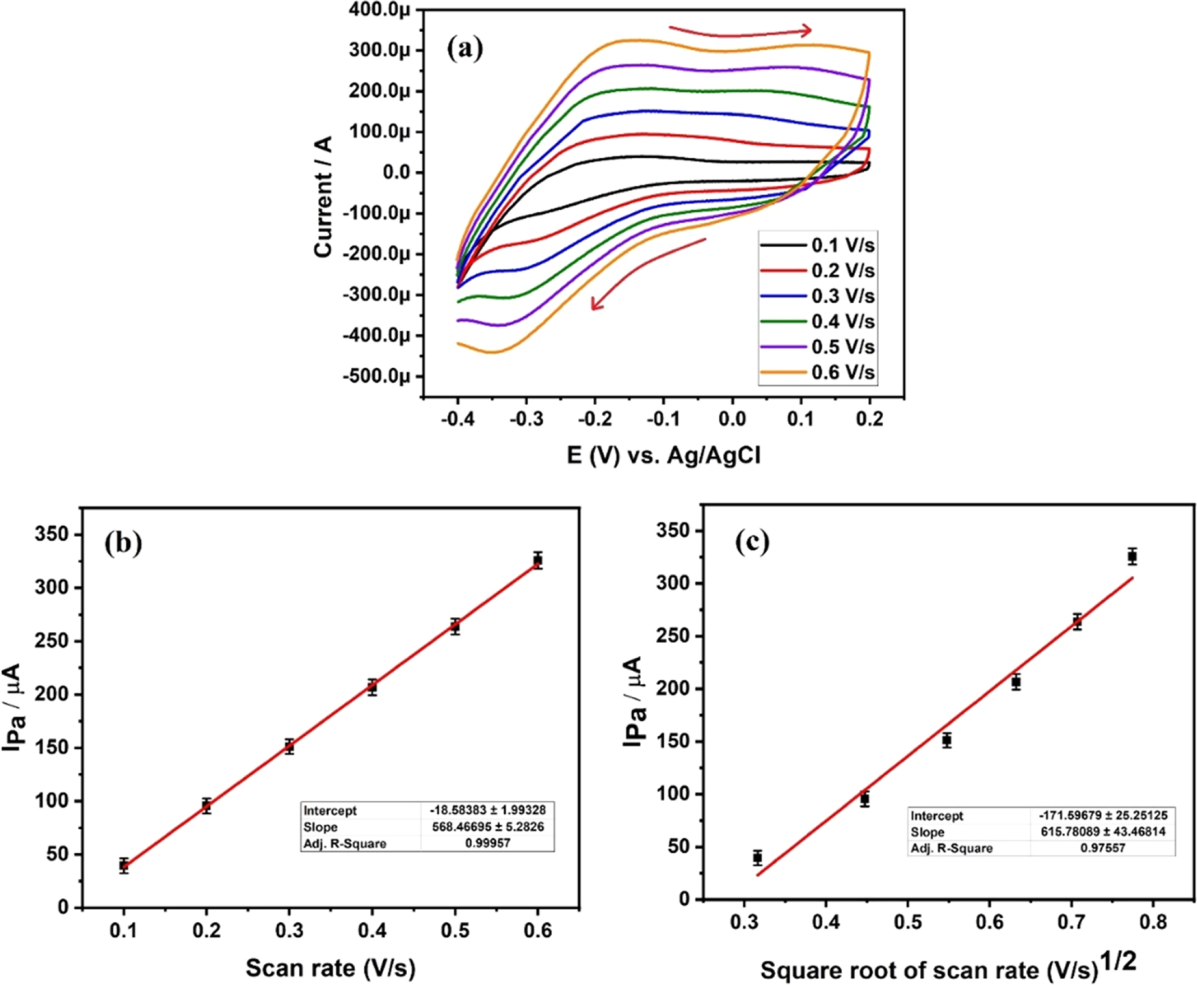
(a) CV curves of 0.1 mM MB at a scan rate from 0.1 to 0.6 V/s on the surface of 2 mg of DSS-MCPE, (b) Graph of Ipa vs scan rate, and (c) Ipa vs square root of scan rate.

Exploring electrochemical oxidation at various scan rates is essential for understanding electrode reactions.^56−58^Figure 7a illustrates the cyclic voltammetry (CV) curve of the MB analyte during electrochemical oxidation at scan rates ranging from 0.1 to 0.6 V/s. The graph affirms that the scan rate exhibits a linear increase in Ipa without alteration of the anodic peak potential. This linearity in Ipa is ascribed to the swift movement of electrons between the 0.1 mM MB analyte and the 2 mg of DSS-MCPE surface. It is complemented by the strong binding of MB molecules to the electrode surface, facilitating significant electronic coupling.^59^ Furthermore, Figure 7b,c depicts graphs of Ipa against scan rate and Ipa against square root of the scan rate, respectively. The correlation coefficients for Figure 7b,c were calculated to be 0.9995 and 0.9755, respectively, indicating that the electrode reaction in both cases is diffusion-controlled.

#### 3.3.4 Effect of MB Concentration on Its Electrochemical Oxidation

**Figure 8:**
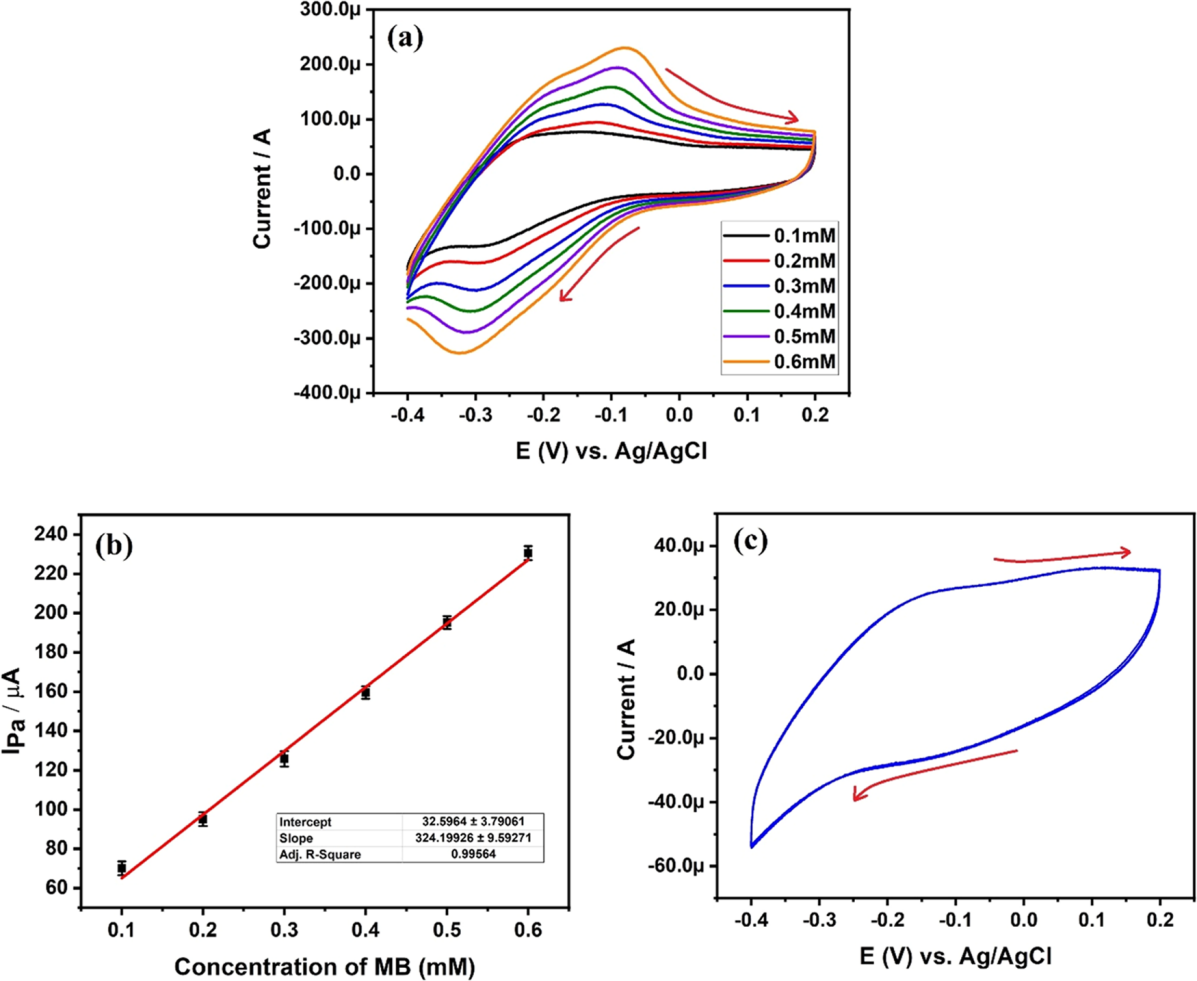
(a) CV curves of different concentrated MB solution and their electrochemical oxidation at 2 mg of DSS-MCPE, (b) plot of *I*_pa_ versus concentration of MB, and (c) 5 cycles of blank voltammogram.

In Figure 8a, the cyclic voltammogram of the MB analyte is depicted at different concentrations, ranging from 0.1 to 0.6 mM MB, in pH 8 PBS at a scan rate of 0.1 V/s. The graph affirms an increase in Ipa from 77.16 to 230.91 μA with the ascending concentrations of MB from 0.1 to 0.6 mM, respectively. This linear augmentation in Ipa is ascribed to the elevated number of MB molecules and the improved electron mobility at higher MB concentrations. Additionally, a slight shift of the oxidation peak potential toward the positive potential is noted with the escalating MB concentrations. This positive potential shift is indicative of the accelerated rate of the electrochemical reaction due to the increased concentrations of MB.

Figure 8b presents a plot of Ipa recorded at different MB concentrations, showcasing a linear increase from 0.1 to 0.6 mM MB with a correlation coefficient of 0.9956. At higher concentrations of the analyte, a robust interaction of MB molecules at the interface of the fabricated 2 mg DSS-MCPE can be anticipated.

Limit of detection (LD) and limit of quantification (LQ) of the electrode were determined using the blank voltammogram (*c*) using eqs 3 and 4 as follows^49^
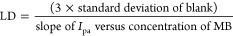
3
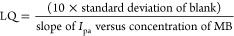
4Calculated values of LD and LQ of 2 mg DSS-MCPE were found to be 0.222 × 10^–8^ and 0.74 × 10^–8^ M, respectively.

#### 3.3.5 Effect of Interferents

**Figure 9:**
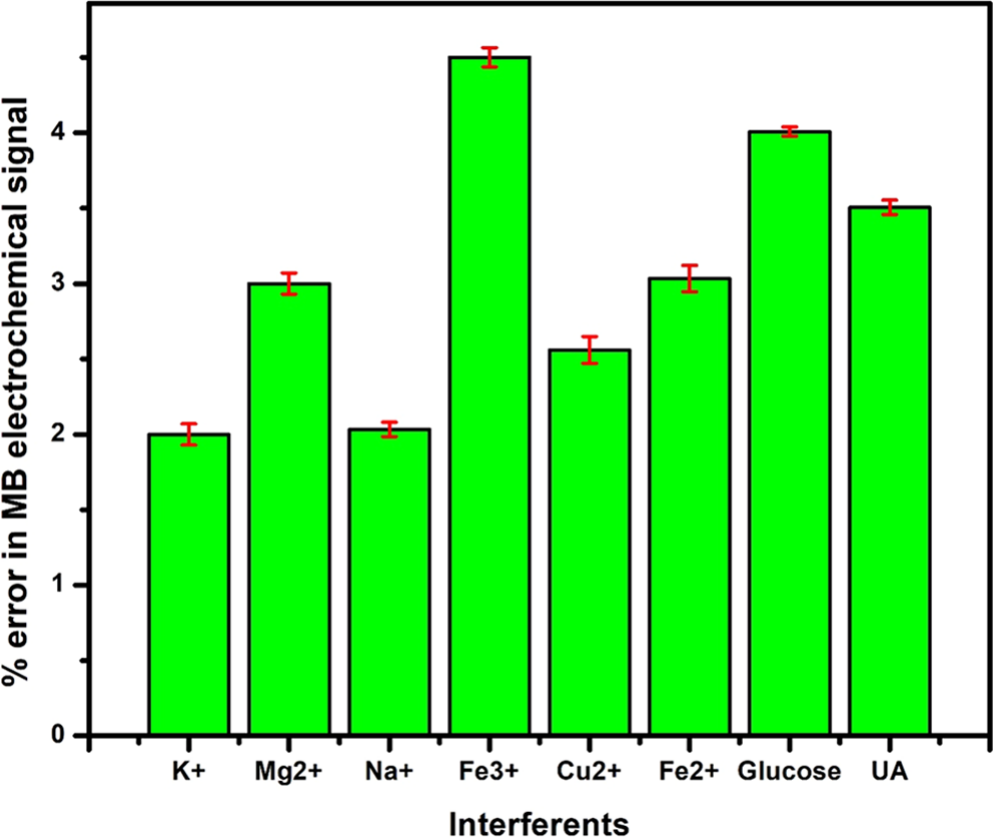
Plot of interferents versus % of error in the electrochemical signal of MB.

The electrochemical oxidation of the 0.1 mM MB analyte on 2 mg of DSS-MCPE was conducted in the presence of interfering metal ions and a few biomolecules. Metal ions such as Na^+^, Cu^2+^, Fe^2+^, Mg^2+^, Fe^3+^, and K^+^, and bioactive molecules like glucose and uric acid were employed as interfering agents to assess their impact on both the oxidation peak current and potential of the 0.1 mM MB during its electrochemical reaction. Notably, no significant increase or decrease, positive or negative shift, was observed in both the peak current and potential of the MB analyte, even in the presence of the aforementioned interfering substances. The electrochemical signal of MB exhibited very slight variations, measuring less than ±5%, as illustrated in Figure 9. This type of investigation into interferences is crucial as it directly reflects the stability, sensitivity, and selectivity of electrodes. In our current study, the recorded variation in the oxidation peak current was less than ±5%, confirming that the fabricated 2 mg of DSS-MCPE demonstrated excellent selectivity and sensitivity, even in the presence of various interfering metal ions and bioactive molecules.

#### 3.3.6 Repeatability, Stability, and Reproducibility of 2 mg of DSS-MCPE

To assess electrode efficiency, a thorough examination of repeatability, stability, and reproducibility was carried out in this study. Electrochemical reactions involving 0.1 mM methylene blue (MB) were conducted in pH 8 phosphate-buffered saline (PBS) at a scan rate of 0.1 V/s to scrutinize the repeatability, stability, and reproducibility of the fabricated 2 mg of DSS-MCPE. Repeatability and reproducibility tests were executed at least five times each, involving changes in both the electrode and electrolyte for every iteration. The calculated relative standard deviation values for reproducibility and repeatability were found to be 2.58 and 2.16%, respectively. These values, significantly lower than expected, suggest that our fabricated 2 mg of DSS-MCPE is well suited for the electrochemical determination of 0.1 mM MB, demonstrating minimal alterations in the oxidation peak current. Essentially, the electrochemical determination using our fabricated electrode can be consistently repeated and reproduced with a minimal deviation from the original oxidation peak current value.

Furthermore, the stability of the fabricated 2 mg of DSS-MCPE was evaluated by subjecting it to 50 continuous cycles of detecting 0.1 mM MB in a pH 8 solution. The oxidation peak currents recorded for the 1st and 50th cycles were considered for stability evaluation. The calculated stability value of 96.51% signifies the excellent stability demonstrated by the fabricated 2 mg of DSS-MCPE during the electrochemical determination of 0.1 mM MB.

## 4 Conclusions

A carbon paste electrode modified with super duplex stainless steel (DSS-MCPE) was successfully developed for the efficient detection of methylene blue dye in wastewater. Varied concentrations of DSS-MCPE (0, 2, 4, 6, 8, and 10 mg) were prepared, with the 2 mg variant demonstrating a substantial maximum current response of 72.22 μA during the electrochemical oxidation of 0.1 mM methylene blue at pH 8. In comparison, a bare carbon paste electrode (BCPE) displayed a considerably lower oxidation peak current of only 8.26 μA. The heightened current response of DSS-MCPE can be ascribed to its augmented surface area, surface roughness, and porosity, as is evident in SEM micrographs. The cyclic voltammogram curve illustrates a linear increase in the anodic peak current with an augmentation in the scan rate, while the anodic peak potential remains constant. A correlation coefficient of less than 1 signifies a diffusion-regulated electrode reaction. Further analysis of the voltammogram reveals a linear increase in the oxidation peak current (from 77.16 to 230.91 μA) as methylene blue concentrations increase from 0.1 to 0.6 mM. This linear correlation suggests that the electrode reaction is diffusion-regulated, with an upsurge in MB molecules promoting enhanced electron contact between the analyte and the electrode surface. To assess the electrode’s selectivity, various metal ions (Na^+^, Cu^2+^, Fe^2+^, Mg^2+^, Fe^3+^, K^+^) and bioactive molecules (glucose and uric acid) were introduced as interfering materials. Notably, there were no significant positive or negative shifts in both the peak current and potential of the MB analyte, affirming the robust selectivity of DSS-MCPE. Moreover, DSS-MCPE exhibited remarkable stability even after 50 cycles of continuous detection of 0.1 mM methylene blue at pH 8, confirming its enduring performance under continuous electrochemical testing. To further extend the impact of this sensing strategy, emphasis should be placed on translating the methodology into portable devices for point-of-care diagnosis. Considering the inherent advantages of the modified electrode, such as enhanced stability and sensitivity, the integration into portable platforms holds promise for rapid and on-site analysis. Future work will focus on refining the fabrication techniques, optimizing device portability, and addressing practical considerations to enable the seamless transition of this electrochemical sensing strategy into real-world point-of-care applications.
